# Triglyceride-glucose index is associated with recurrent revascularization in patients with type 2 diabetes mellitus after percutaneous coronary intervention

**DOI:** 10.1186/s12933-023-02011-2

**Published:** 2023-10-21

**Authors:** Qiang Chen, Shiqiang Xiong, Zhen Zhang, Xiuqiong Yu, Yingzhong Chen, Tao Ye, Siqi Yang, Lingyao Qi, Xu Chen, Hanxiong Liu, Jingang Zheng, Lin Cai

**Affiliations:** 1 Graduate School of Peking Union Medical College, Chinese Academy of Medical Sciences and Peking Union Medical College, Beijing, 100029 China; 2 Department of Cardiology, The Third People’s Hospital of Chengdu, Affiliated Hospital of Southwest Jiaotong University, Chengdu, 610014 Sichuan China; 3 Department of Cardiology, China-Japan Friendship Hospital, Beijing, 100029 China

**Keywords:** The triglyceride-glucose index, Type 2 diabetes mellitus, Insulin resistance, Percutaneous coronary intervention, Recurrent revascularization

## Abstract

**Background:**

The Triglyceride-glucose (TyG) index, as a surrogate marker of insulin resistance, is independently associated with the severity of coronary artery lesions and the prognosis of coronary heart disease. The investigation aimed to explore the relationship between the TyG index and recurrent revascularization in individuals with type 2 diabetes mellitus (T2DM) resulting from the progression of lesions or in-stent restenosis (ISR) after percutaneous coronary intervention (PCI).

**Method:**

A total of 633 patients who met the inclusion and exclusion criteria were enrolled and divided into three groups based on the tertiles of the TyG index. The primary endpoint was recurrent revascularization resulting from the progression of lesions or ISR. All-cause death was considered as the competing risk event. Competing risk analysis and Cox regression analysis for predicting recurrent revascularization after PCI were conducted stepwise. Variables were standardized to make the hazard ratio (HR), subdistribution hazard ratio (SHR) and corresponding 95% CI more consistent prior to being used for fitting the multivariate risk model. The predictive ability of the TyG index was evaluated using several measures, including the ROC curve, likelihood ratio test, Akaike’s information criteria, category-free continuous net reclassification improvement (cNRI > 0), and integrated discrimination improvement (IDI). Internal validation was conducted through bootstrapping with 1000 resamples.

**Results:**

During a median follow-up period of 18.33 months, a total of 64 (10.11%) patients experienced recurrent revascularization, including 55 cases of lesion progression and 9 cases of in-stent restenosis. After controlling for competitive risk events, the TyG index was independently associated with a higher risk of recurrent revascularization [SHR:1.4345, (95% CI 1.1458–1.7959), P = 0.002]. The likelihood ratio test and Akaike’s information criteria showed that the TyG index significantly improves the prognostic ability. Additionally, adding the TyG index improved the ability of the established risk model in predicting recurrent revascularization, indicated by a C-index of 0.759 (95% CI 0.724–0.792, P < 0.01), with a cNRI > 0 of 0.170 (95% CI 0.023–0.287, P < 0.05), and an IDI of 0.024 (95% CI 0.009–0.039, P = 0.002). These results remained consistent when the models containing TyG index were confirmed using an internal bootstrap validation method.

**Conclusion:**

The findings highlight the potential of the TyG index as a predictor of recurrent revascularization. Lesion progression emerged as the primary contributor to recurrent revascularization instead of in-stent restenosis. The incorporation of the TyG index into risk prediction models is likely to be beneficial for accurate risk stratification in order to improve prognosis.

**Supplementary Information:**

The online version contains supplementary material available at 10.1186/s12933-023-02011-2.

## Introduction

With significant advancements in the technological and procedural evolution of percutaneous coronary intervention (PCI), along with improvements in medical therapies such as dual antiplatelet therapy (DAPT) and intensive lipid-lowering therapy, there has been a notable decrease in mortality resulting from primary ischemic events for patients with coronary heart disease (CHD) over the past decade [1]. However, due to the increasing number of patients undergoing percutaneous coronary intervention (PCI), a significant proportion of patients are still at a high risk of experiencing recurrent revascularization due to in-stent restenosis (ISR) or lesion progression. This risk is particularly pronounced among patients with type 2 diabetes mellitus (T2DM) [2]. The original factors and underlying pathophysiological mechanisms contributing to recurrent revascularization are multifaceted and encompass various aspects, including lesion anatomy, interventional procedures, inflammatory molecules, cardiometabolic risk factors (CMRFs), nutritional status, individual genetic factors, and other variables [3]. Risk stratification based on fully evaluating various risk factors of patients may have great significance to tailor individualized secondary prevention measures and implement precise follow-up management, which is conducive to early detection of progressive lesions and reduction of recurrent revascularization [4].

Insulin resistance (IR), a well-established mediator of cardiovascular disease, may contribute to the development of atherosclerotic plaque formation by triggering oxidative stress and promoting an inflammatory response [5–8]. Accordingly, there is substantial speculation regarding the significant association recurrent revascularization resulting from ISR or progression of lesion among patients with T2DM underwent PCI. The triglyceride-glucose (TyG) index, which has been demonstrated a surrogate marker of IR that is comparable to the homeostasis model assessment of IR (HOMA-IR) [9, 10], may contribute to an increased prevalence of adverse cardiovascular outcomes in the post-PCI population regardless of the presence of diabetes [11–13]. Currently, there is limited research specifically examining the association between the TyG index and recurrent revascularization in patients with T2DM who underwent PCI.

The purpose of this study was to investigate this knowledge gap by examining the relationship between the TyG index and recurrent revascularization driven by the progression of lesion or ISR in the population of individuals with T2DM who underwent PCI.

## Methods

### Study population

Patients diagnosed with CHD and T2DM who underwent regular reviews following successful PCI at the Third People’s Hospital of Chengdu (Sichuan, China), which is affiliated with Southwest Jiaotong University, were retrospectively enrolled in the study from July 2018 to December 2020. The following exclusion criteria were as follows: (1) a history of coronary artery bypass grafting (CABG); (2) death during hospitalization; (3) critical structural heart disease requiring intervention; (4) stage of refractory end-stage heart failure (LVEF < 30%); (5) advanced malignant tumors with short life expectancy; (6) severe liver, renal, or respiratory function insufficiency; (7) incomplete critical medical data exceeding 10%. The study was conducted ethically and in accordance with the principles of the Declaration of Helsinki. The Ethics Committee of the Third People’s Hospital in Chengdu approved the study. All participants in the study gave their informed consent either in written or oral form. Ultimately, a total of 633 patients were included in the final analysis (Additional file 1: Fig. S1).

### Data collection and definitions

Demographic data, previous medical history, laboratory results, and procedural information, including echocardiography and angiographic evaluation results, were collected from the electronic medical record system of the Third People’s Hospital of Chengdu by trained physicians who were blinded to the purpose of the stud. Body mass index (BMI) was calculated as weight (kg)/[height squared (m^2^)]. T2DM was diagnosed based on a history of diabetes mellitus previously diagnosed by a professional physician, a definite medical history previously diagnosed by a professional physician, or the symptoms of diabetes mellitus with casual blood glucose ≥ 11.1 mmol/L, fasting blood glucose (FBG) ≥ 7.0 mmol/L, and/or 2-h blood glucose ≥ 11.1 mmol/L in the 75 g oral glucose tolerance test [14]. Hypertension was defined as at least three blood pressure measurements showing systolic blood pressure ≥ 140 and/or diastolic blood pressure ≥ 90 mmHg, and/or currently receiving antihypertensive treatments [15]. In addition, based on angiographic assessment, the presence of significant diameter stenosis (≥ 50%) in two or more vessels was defined as multivessel disease (MVD) [16], and completed obstruction of a native coronary artery for ≥ 3 months was defined as chronic total occlusion (CTO) [17]. Left ventricular ejection fraction (LVEF) was determined by the two-dimensional modified Simpson’s methods [18]. A web-based online calculation tool (http://syntaxscore.com/) was used to assess the baseline SYNTAX score (bSS) and residual SYNTAX score (rSS) after the last revascularization by two independent interventionalists who were blinded to baseline clinical characteristics and clinical outcomes [19].

Blood samples were obtained from peripheral veins of patients following an overnight fast of > 8 h while hospitalized based on clinical indications. Plasma concentrations of total cholesterol (TC), triglycerides (TG), low-density lipoprotein-C (LDL-C), high-density lipoprotein-C (HDL-C), fasting blood glucose (FBG), serum creatinine, brain natriuretic peptide (BNP), and cardiac troponin T (cTnT) concentrations were determined using standard laboratory methods in the central laboratory. The TyG index was calculated as the following formula: ln [fasting TG (mg/dL) × FBG (mg/dL)/2] [9].

### Follow-up and endpoints

The clinical follow-up appointments were initially scheduled at 1, 3, 6, and 12 months after hospital discharge, followed by annual follow-up visits, conducted by trained personnel who were unaware of the study details, either via phone calls or in-person clinic visits. The primary endpoint was defined as recurrent revascularization by PCI or coronary artery bypass grafting (CABG) after indexing procedures, driven by progression of lesion or ISR during follow-up. In line with previous guidelines, ISR was condemned when significant diameter stenosis (≥ 50%) was observed inside the stent or involving its 5-mm edges. Definition of progression of lesion was that progression of lesion with grade of stenosis < 70% at index angiography, ultimately resulting in unplanned ischemia-driven revascularization at the time of angiographic follow-up. Staged revascularization within 3 months at the decision of the cardiologist was not considered a primary endpoint. All-cause death, which encompasses deaths resulting from cardiac or non-cardiac factors, is considered a significant competing event. The information regarding endpoint event was obtained through telephone contact with the patients or their family members by trained personnel blinded to the baseline characteristics and further ascertained by careful review of the corresponding medical records and angiographic images by experienced cardiologists.

### Statistical analysis

Continuous variables were described as the mean ± standard deviation (SD) or the median with interquartile range depending on the normality of the data distribution, and comparisons between groups were performed with ANOVA test or Kruskal–Wallis H test, respectively. Categorical variables were presented as frequencies and percentages, and comparisons between groups were carried out using the chi-square (χ^2^) test or Fisher’s exact test. Participants were stratified into three groups based on the TyG index tertiles: T1 (TyG index < 8.85, n = 210), T2 (8.85 ≤ TyG index < 9.28, n = 211) and T3 (TyG index ≥ 9.28, n = 212). All statistical analyses in the present study were performed with SPSS 24.0 (IBM, Armonk, New York), R Programming Language 4.0.2, Stata/MP 16.0 software and MedCalc19.1 (MedCalc software, Belgium). All tests were 2-sided, and P < 0.05 was considered statistically significant.

Competing risk models were used to analyze the data, with recurrent revascularization as the outcome event and all-cause deaths as the competing event. Univariate competing risk analysis was conducted to plot cumulative incidence function (CIF) curves for the endpoint and to compare differences between groups using the Fine-Gray test. Variables with statistical significance (P < 0.05) were screened for inclusion in the multivariate competing risk analysis. Meanwhile, Univariate and multivariate Cox regression analysis were also conducted stepwise. Variables have been standardized to make the hazard ratio (HR), subdistribution hazard ratio (SHR) and corresponding 95% CI more consistent before being used for fitting the multivariate risk model. Predictive capacities of variables were assessed respectively by the aera under the curve (AUC) and Receiver Operating Characteristic (ROC) curves, and compared each other using the DeLong’s test.

χ^2^ likelihood ratio tests were carried out to determine whether the established logistic regression model incorporating TyG index, TG, FBG or HbA1c provided a significantly better fit. The comparison of nested and non-nested models, including the established risk model or its combination with the TyG index, HbA1c, TG or FBG, was performed by calculating corrected Akaike’s information criterion (AICc), delta-AICc (δAICc), and AICc weights (AICcWt) to estimate the probability that a given model is the best fitting model among those studied bootstrap method (with 1000 bootstrapped samples) was carried out to validate the effectiveness of the model.

The degree of discrimination of the model, which refers to its ability to distinguish between individuals who have an endpoint event and those who have not, was measured using the relatively corrected C-index. Calibration curves were also plotted to evaluate the accuracy of the model prediction, that is, the consistency between the predicted probability of the event and the actual probability of occurrence. Additionally, C-statistics, category-free continuous net reclassification improvement (cNRI > 0), and integrated discrimination improvement (IDI) were compared between models to determine whether adding the TyG index to the established risk model had incremental predictive value for primary outcomes. The event NRI (NRIe) was defined as the net percentage of individuals in which the event of interest is correctly classified with a higher predicted risk and non-event NRI (NRIne) as the net percentage of individuals without the event of interest that are correctly assigned a lower predicted risk.

## Results

### Baseline characteristics of the total population

A total of 633 patients who met the inclusion and exclusion criteria, with an average age of 68.02 ± 10.75 years, were included in the final analysis ultimately. Patients were stratified into three groups based on the tertiles of TyG index. The baseline characteristics of the total population are presented in Table 1. The mean TyG index values among the three groups were 8.50 ± 0.32, 9.05 ± 0.13, and 9.67 ± 0.38, respectively. There were significant differences (P < 0.05) among the three groups in terms of Age, BMI, HR, FBG, HbA1c, TG, TC, HDL-C, LDL-C, LM and CTO. Patients with a higher TyG index tended to be younger (66.56 ± 10.91 years) on average, had a higher BMI, and showed higher plasma concentrations of FBG, HbA1c, TG, TC, and LDL-C, as well as lower plasma HDL-C, compared to patients in the lower group. No significant differences were found between the three groups in regard to the other indicators (P > 0.05).

**Table 1:** Baseline characteristics of patients stratified by the TyG index tertiles

Variable	T1 (n = 210)	T2 (n = 211)	T3 (n = 212)	P value
Age, years	70.28 ± 10.53	67.50 ± 10.46	66.31 ± 10.91	< 0.001
Female, n (%)	67 (31.9)	71 (33.6)	70 (33.0)	0.928
BMI, kg/m^2^	24.24 ± 2.93	24.99 ± 2.85	25.45 ± 3.40	< 0.001
Smoking, n (%)	90 (42.9)	95 (45.0)	99 (46.7)	0.729
Previous PCI, n (%)	28 (13.3)	16 (7.6)	24 (11.3)	0.154
COPD, n (%)	11 (5.2)	8 (3.8)	9 (4.2)	0.762
Hypertension, n (%)	156 (74.3)	165 (78.2)	156 (73.6)	0.495
AF, n (%)	19 (9.0)	13 (6.2)	20 (9.4)	0.408
Previous stroke, n (%)	15 (7.1)	8 (3.8)	13 (6.1)	0.313
SBP, mmHg	133.19 ± 21.80	134.00 ± 22.87	134.79 ± 21.21	0.459
HR, bpm	77.51 ± 13.86	77.36 ± 14.72	81.45 ± 15.72	0.006
cTnT, pg/mL	25.90 (13.80, 550.40)	28.66 (13.00, 502.10)	49.17 (14.00, 459.30)	0.388
BNP, pg/mL	97.90 (40.55, 304.25)	99.60 (34.70, 328.80)	137.18 (45.93, 485.15)	0.132
Scr, µmol/L	79.85 (66.70, 98.35)	76.00 (64.00, 92.70)	78.55 (63.03, 101.20)	0.248
Uric acid, µmol/L	369.76 ± 117.54	386.24 ± 125.92	379.99 ± 127.59	0.387
FBG, mmol/L	6.70 ± 2.06	8.38 ± 2.49	10.28 ± 3.85	< 0.001
HbA1c	7.00 ± 1.40	7.74 ± 1.78	8.04 ± 1.78	< 0.001
TG, mmol/L	1.01 ± 0.29	1.38 ± 0.39	2.22 ± 0.99	< 0.001
TC, mmol/L	3.98 ± 1.13	4.40 ± 1.28	4.78 ± 1.27	< 0.001
HDL-C, mmol/L	1.17 ± 0.30	1.09 ± 0.28	1.12 ± 0.30	0.012
LDL-C, mmol/L	2.37 ± 0.78	2.68 ± 0.89	2.93 ± 0.90	< 0.001
LVEF	55.01 ± 9.39	55.43 ± 9.37	53.28 ± 10.23	0.053
AMI, n (%)	89 (42.4)	89 (42.2)	106 (50.0)	0.183
Diagnosis, n (%)				0.429
CCS	33 (15.7)	35 (16.6)	22 (10.4)	
UA	88 (41.9)	87 (41.2)	84 (39.6)	
NSTEMI	48 (22.9)	43 (20.4)	53 (25.0)	
STEMI	41 (19.5)	46 (21.8)	53 (25.0)	
Aspirin, n (%)	204 (97.1)	206 (97.6)	203 (95.3)	0.519
P_2_Y_12_ receptor inhibitor, n (%)	208 (99.0)	210 (99.5)	208 (98.1)	0.368
Statins, n (%)	203 (96.7)	206 (97.6)	209 (98.6)	0.432
β-Blockers, n (%)	145 (69.0)	145 (68.7)	162 (76.4)	0.141
ACEI/ARB, n (%)	100 (47.6)	108 (51.2)	112 (52.8)	0.550
Diuretics, n (%)	39 (18.6)	47 (22.3)	53 (25.0)	0.278
Insulin, n (%)	45 (21.4)	62 (29.4)	60 (28.3)	0.133
Oral hypoglycemic agents, n (%)	145 (69.0)	156 (73.9)	149 (70.3)	0.516
MVD, n (%)	151 (71.9)	154 (73.0)	168 (79.2)	0.172
LM, n (%)	11 (5.2)	24 (11.4)	10 (4.7)	0.013
Calcified lesions, n (%)	38 (18.1)	39 (18.5)	42 (19.8)	0.894
Thrombosis, n (%)	19 (9.0)	16 (7.6)	23 (10.8)	0.507
Long lesion, n (%)	125 (59.9)	130 (61.6)	141 (66.5)	0.314
CTO, n (%)	41 (19.5)	39 (18.5)	59 (27.8)	0.039
Number of stents	1.51 ± 0.92	1.51 ± 0.94	1.58 ± 0.96	0.643
Length of stents, mm	39.18 ± 26.43	40.05 ± 28.58	43.20 ± 29.07	0.301
TyG index	8.50 ± 0.32	9.05 ± 0.13	9.67 ± 0.38	< 0.001
bSS	14.00 (8.00, 21.00)	16.00 (9.00, 21.50)	15.75 (10.00, 21.38)	0.379
rSS	3.00 (0.00, 8.00)	5.00 (0.00, 9.00)	4.00 (1.00, 9.00)	0.286

Data are presented as mean ± SD, median (IQR) or n (%)

*BMI* body mass index, *PCI* percutaneous coronary intervention, *COPD* chronic obstructive pulmonary disease, *AF* atrial fibrillation, *SBP* systolic blood pressure, *HR* heart rate, *BNP* brain natriuretic peptide, *Scr* serum creatinine, *FBG* fasting blood glucose, *TG* triglyceride, *TC* total cholesterol, *HDL-C* high density lipoprotein cholesterol, *LDL-C* low density lipoprotein cholesterol, *LVEF* left ventricular ejection fraction, *AMI* acute myocardial infarction, *CCS* chronic coronary syndrome, *UA* unstable angina, *STEMI* ST-segment elevation myocardial infarction, *NSTEMI* non-ST-segment elevation myocardial infarction, *ACEI/ARB* angiotensin converting enzyme inhibitor/angiotensin receptor blocker, *MVD* multivessel disease, *LM* left main disease, *CTO* chronic total occlusion, *TyG index* the triglyceride-glucose index, *bSS* baseline SYNTAX score, *rSS* residual SYNTAX score, *T1* TyG index < 8.85, *T2* 8.85 ≤ TyG index < 9.28, *T3* TyG index ≥ 9.28

**Fig. 1:**
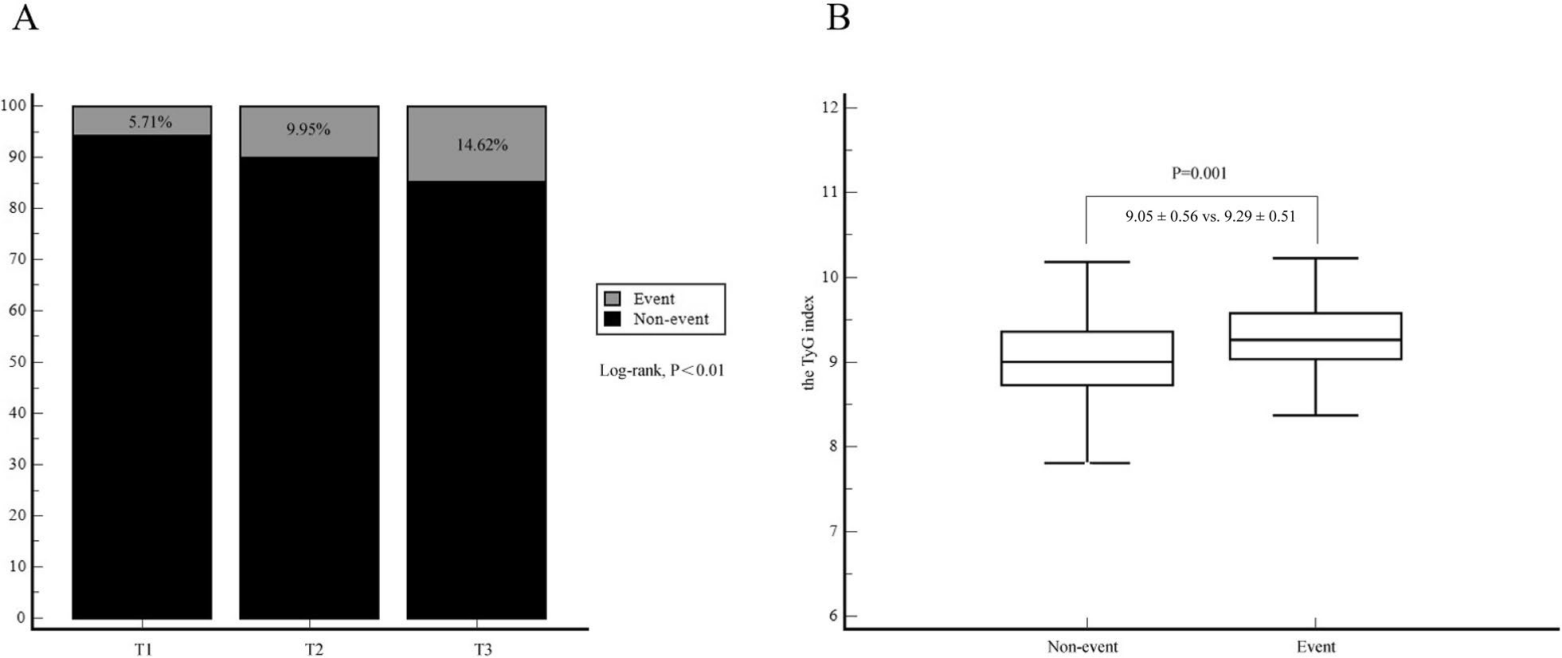
The effects of the TyG index on the incidence of recurrent revascularization (**A**) and the discrepancy in TyG index levels between the groups with and without events (**B**) within the cohort of enrolled participants. Event refers to the incidence of recurrent revascularization, Non-event refers to patients without recurrent revascularization. *T1* TyG index < 8.85, *T2* 8.85 ≤ TyG index < 9.28, *T3* TyG index ≥ 9.28

During a median follow-up of 18.83 months (interquartile range, 14.58 to 22.88 months), we recorded 64 (10.11%) cases of recurrent revascularization events, including 55 cases of lesion progression and 9 cases of in-stent restenosis. In subsequent analysis, we discovered that cases of unplanned revascularization were primarily caused by the progression of lesions rather than in-stent restenosis (Additional file 1: Fig. S2). Patients with higher TyG index exhibited a significantly higher incidence of recurrent revascularization compared to those with lower TyG index [12 (5.71%) vs. 21 (9.95%) vs. 31 (14.62%), P < 0.01] (Fig. 1A, B).

### Competing risk analysis and Cox regression analysis for predicting recurrent revascularization after PCI

**Fig. 2:**
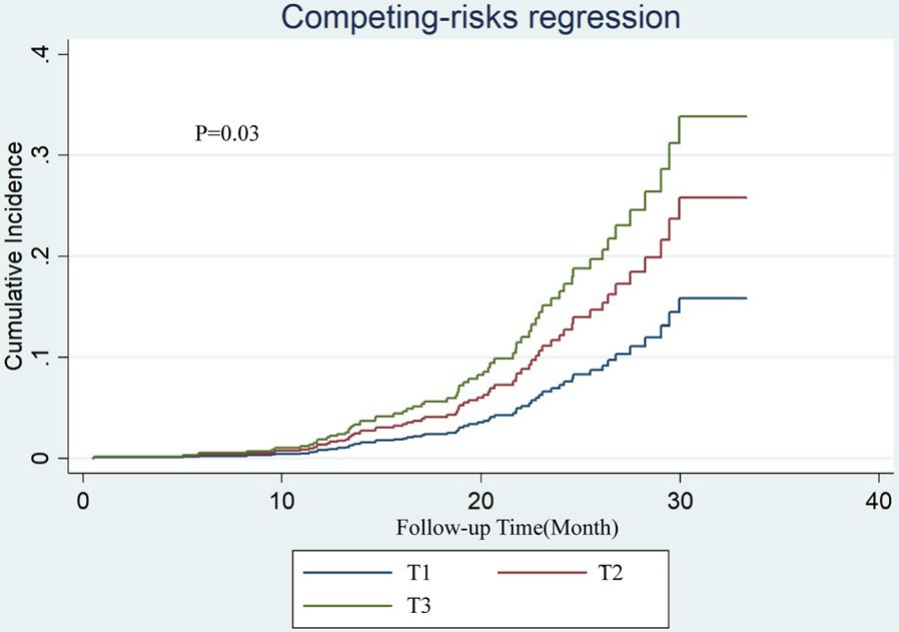
Competing risk analysis of different TyG index tertiles. After controlling for competitive risk events, significant differences persist in the cumulative risk of recurrent revascularization among the three groups (P = 0.03). *T1* TyG index < 8.85, *T2* 8.85 ≤ TyG index < 9.28, *T3* TyG index ≥ 9.28

**Fig. 3:**
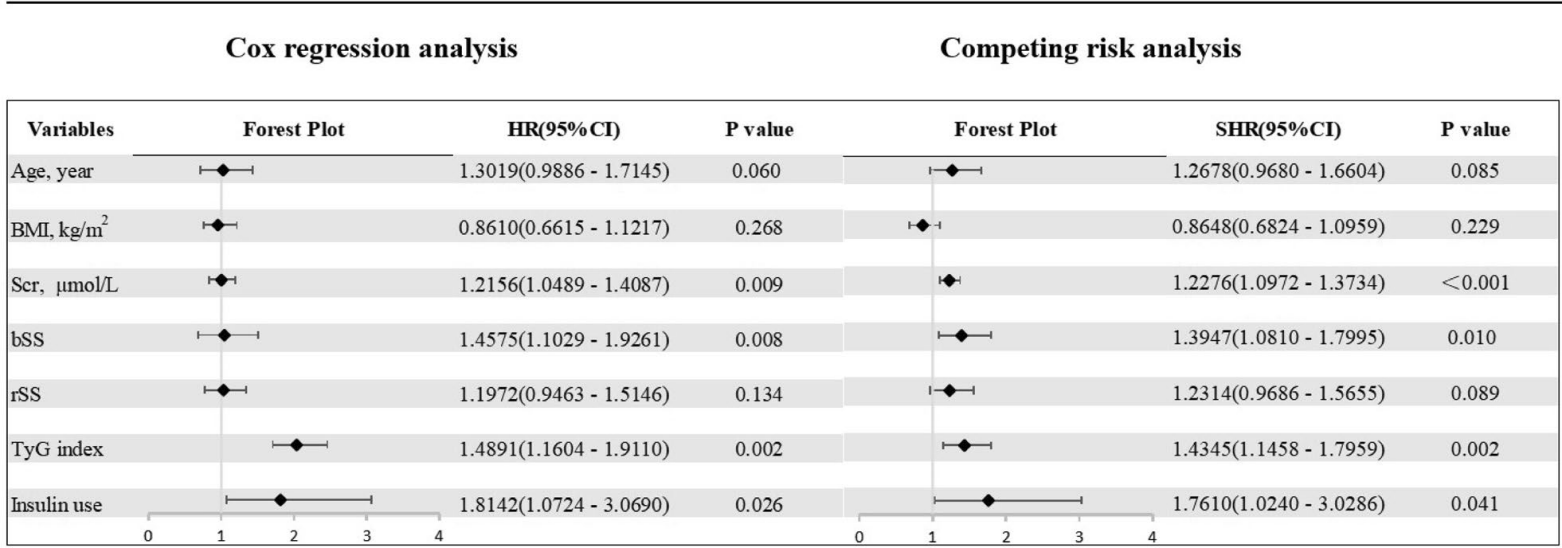
Forest plot illustrating the association between the TyG index and recurrent revascularization after PCI in patients with T2DM using multivariable Cox regression analysis and multivariable competing risk analysis. *T2DM* type 2 diabetes mellitus, *TyG index* triglyceride-glucose index, *BMI* body mass index, *PCI* percutaneous coronary intervention, *bSS* baseline SYNTAX score, *rSS* residual SYNTAX score, *HR* hazard ratio, *SHR* subdistribution hazard ratio, *HR* hazard ratio, *CI* confidence interval. Variables have been standardized to make the HR, SHR and corresponding 95% CI more consistent prior to being used for fitting the statistical model. HR, as well as SHR, represent the risk ratio associated with a one-unit increase in the standard deviation of the variable

A total of 35 (5.53%) cases of all-cause death as the competing event was recorded. After controlling for competitive risk events, significant differences persist in the cumulative risk of recurrent revascularization among the three groups (Fine-Gray test, P < 0.03, Fig. 2). Univariate competing risk analysis revealed that BMI (P = 0.027), serum creatinine (P < 0.001), bSS (P < 0.001), rSS (P < 0.001), TyG index (P = 0.005), insulin use (P = 0.003) were associated with recurrent revascularization after excluding the interference of competing events (Additional file 1: Table S1). Univariate statistically significant variables, combined with age, were further included in the multivariate competing risk analysis. The outcome indicated the TyG index was independently associated with a higher risk of recurrent revascularization [SHR: 1.4345, (95% CI 1.1458–1.7959), P = 0.002, Fig. 3].

Furthermore, we also conducted univariate and multivariate Cox proportional hazards regression analyses to identify predictors of recurrent revascularization stepwise (Additional file 1: Table S1). After adjusting for BMI, serum creatinine, bSS, rSS, TyG index and insulin use, it was found that the TyG index was independently associated with a higher risk of recurrent revascularization in patients with diabetes mellitus undergoing PCI [HR: 1.4891, (95% CI 1.1604–1.9110), P = 0.002, Fig. 3].

### The predictive value of the TyG index for recurrent revascularization

**Fig. 4:**
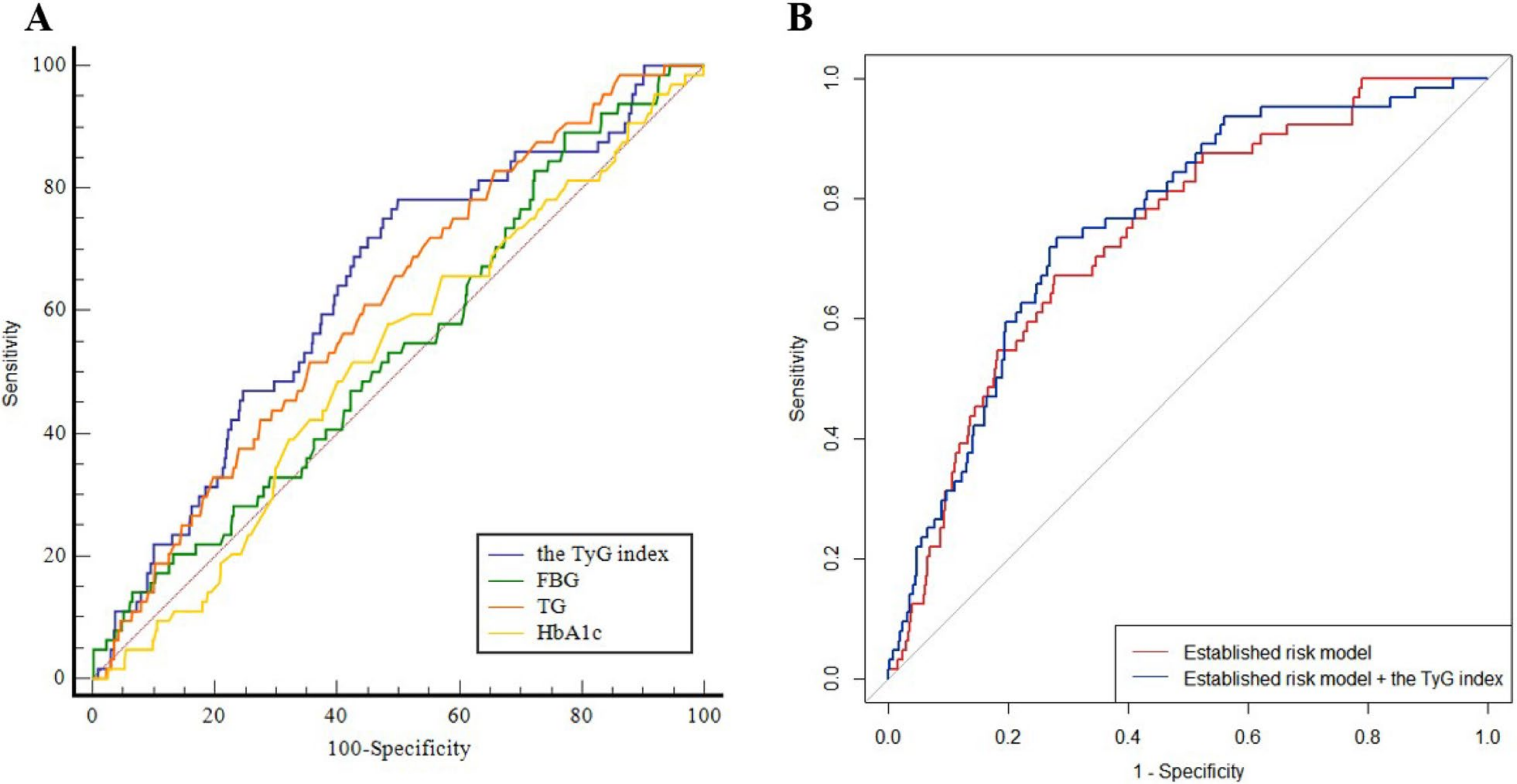
ROC analysis of the TyG index, HbA1c, TG and FBG to predict recurrent revascularization (**A**) and comparison of the C-statistics between the models (**B**). *TyG index* triglyceride-glucose index, *FBG* fasting blood glucose, *TG* triglycerides, *AUC* area under curve. Established risk model included age, BMI, Scr, bSS, rSS and insulin. C-statistics were compared each other using the DeLong’s test. The TyG index as a continuous variable

The area under the receiver operating characteristic curve (AUC) for the TyG index (0.631, 95% CI 0.560–0.702) predicting recurrent revascularization was significantly higher compared to fasting blood glucose (FBG) (0.539, 95% CI 0.466–0.612) (P = 0.0046) and HbA1c (0.520, 95% CI 0.448–0.592) (P = 0.0438). Furthermore, the AUC for the TyG index was greater than that of TG (0.612, 95% CI 0.544–0.680), but this difference was not statistically significant (P = 0.3675) (Fig. 4A) (Additional file 1: Tables S2, S3).

### Incremental effects of the TyG index on the predictive value of recurrent revascularization

The performance of the model after adding the TyG index to the baseline established model in patients is presented in Table 2. The likelihood ratio tests results showed that the addition of the TyG index as a continuous variable significantly improved the model fit (χ^2^ = 11.252, 1 df; P ≤ 0.001). Addition of HbA1c or FBG did not improve the model fit. Additionally, when comparing the baseline established risk model alone, the incorporation of the TyG index into risk prediction models resulted in the lowest corrected AIC (245.88) and highest Akaike’s weight (0.94).

**Table 2:** Akaike’s information criteria and likelihood ratio test to determine the best fitting model for predicting recurrent revascularization

	Akaike’s information criteria				Likelihood ratio test			
	AICc	δAICc	AICcWt	Cum.wt	Model	χ^2^	df	P value
Established risk model	255.08	9.19	0.01	0.99	Established risk model	Ref	Ref	Ref
Established risk Model + TG	252.66	6.78	0.03	0.97	Established risk Model + TG	4.473	1	0.034
Established risk Model + HbA1c	255.95	10.06	0.01	1.00	Established risk Model + HbA1c	1.189	1	0.276
Established risk Model + FBG	254.75	8.87	0.01	0.98	Established risk Model + FBG	2.3823	1	0.123
Established risk Model + TyG	245.88	0.00	0.94	0.94	Established risk Model + TyG	11.252	1	< 0.001

Established risk model included age, BMI, Scr, bSS, rSS and insulin

*TyG index* the triglyceride-glucose index, *TG* triglyceride, *FBG* fasting blood glucose, *AICc* corrected Akaike’s information criterion, *δAICc* delta-AICc, *AICcWt* AICc weights, *Cum.wt* the cumulative weights of AIC

The addition of the TyG index to the baseline model of established risk factors resulted in a significant increase in the C-statistic from 0.739 (95% CI 0.703–0.773) to 0.759 (95% CI 0.724–0.792) (P < 0.01) (Additional file 1: Table S4). It also led to a significant improvement in reclassification as assessed by the cNRI > 0 (0.170, 95% CI 0.023–0.287, P < 0.05), and IDI (0.024, 95% CI 0.009–0.039, P = 0.002). However, adding FBG, TG or HbA1c to the established model did not result in a significant improvement in net reclassification or integrated discrimination for predicting recurrent revascularization during follow-up (Table 3). These results indicate that the model incorporating the TyG index is likely to be the best fitting model and may have a better ability to predict recurrent revascularization compared to FBG, TG, or HbA1c.

**Table 3:** The incremental predictive value of various models evaluated by cNRI > 0 and IDI

	cNRI > 0 (95% CI)	NRIe	NRIne	P value	IDI (95% CI)	P value
Established risk model	Ref	Ref	Ref	Ref	Ref	Ref
Established risk Model + TG	0.061 (− 0.011, 0.260)	0.078	− 0.018	> 0.05	0.008 (0.000, 0.015)	0.044
Established risk Model + HbA1c	− 0.026 (− 0.050, 0.171)	− 0.031	0.005	> 0.05	0.001 (− 0.005, 0.007)	0.707
Established risk Model + FBG	0.043 (− 0.007, 0.178)	0.062	− 0.019	> 0.05	0.006 (− 0.003, 0.014)	0.192
Established risk Model + TyG	0.170 (0.023, 0.287)	0.172	− 0.002	< 0.05	0.024 (0.009, 0.039)	0.002

Established risk model included age, BMI, Scr, bSS, rSS and insulin

*TyG index* the triglyceride-glucose index, *TG* triglyceride, *CI* confidence interval, *FBG* fasting blood glucose, *cNRI > 0* category-free continuous net reclassification improvement, *IDI* integrated discrimination improvement, *NRIe* event NRI, *NRIne* non-event NRI

### The performance of the prediction models evaluated by internal bootstrap validation

**Fig. 5:**
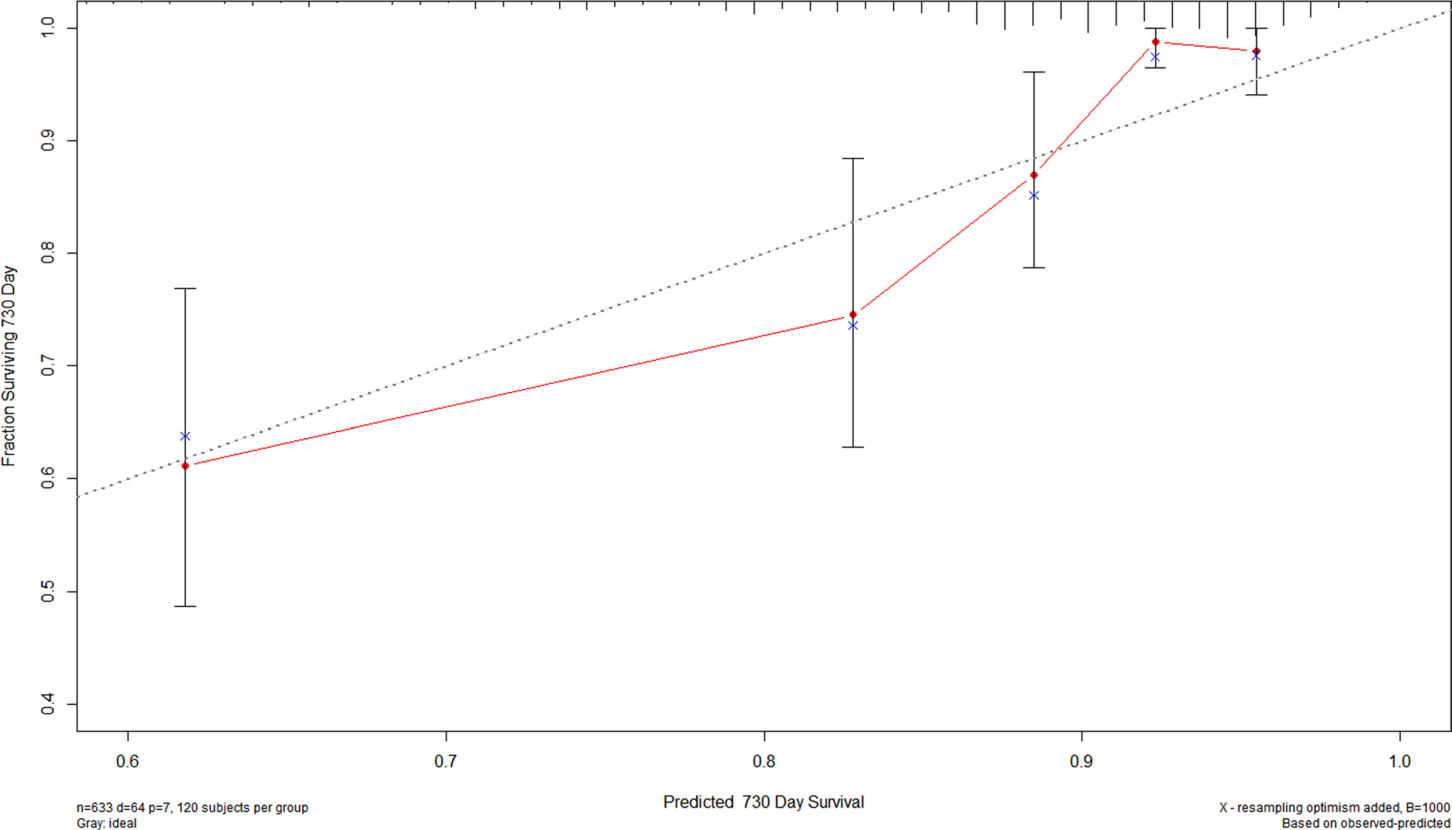
The calibration plots for the adjusted model predicting recurrent revascularization. The x-axis represents the predicted adverse cardiovascular events risk. The y-axis represents the actual adverse cardiovascular events rate. The grey line indicates a perfect prediction by an ideal model. The red solid line indicates the performance of the predicting model, of which a closer fit to the grey line suggests better prediction. The adjusted model refers to the established risk model combined with the TyG index. Established risk model included age, BMI, Scr, bSS, rSS and insulin

The results remained consistent when the models were validated using the internal bootstrap validation method (Additional file 1: Table S4). The bias-corrected C-statistic of the new model adjusted by the TyG index, which was superior to that adjusted by FBG, TG or HbA1c, was significantly higher than that of the unadjusted baseline model of established risk factors for predicting of recurrent revascularization [0.747 (95% CI 0.692–0.793) vs. 0.769 (95% CI 0.718–0.812)] (P < 0.01). The calibration plots for the adjusted model by TyG index predicting recurrent revascularization exhibited excellent agreement between the observed and predicted probabilities (Fig. 5).

## Discussion

The present study demonstrates that the association between the TyG index and the risk of recurrent revascularization resulting from ISR or progression of lesion in patients with T2DM for the first time. The main findings of this study are as follows: (1) patients with a higher TyG index had a higher likelihood of experiencing recurrent revascularization after successful PCI; (2) the TyG index independently predicted recurrent revascularization in the fully adjusted competing risk analysis and Cox regression analysis; (3) the TyG index demonstrated better predictive value for recurrent revascularization compared to FBG, TG, or HbA1c; and (4) The addition of the TyG index improved the capability of established risk model to predict recurrent revascularization. The findings of our study suggest that considering the TyG index can optimize secondary prevention treatment and follow-up strategies for patients with T2DM who have underwent PCI.

The number of patients receiving interventional therapy for coronary heart disease worldwide has been steadily increasing. In the United States, the volume of percutaneous coronary intervention (PCI) procedures rose by 15.8% from 550,872 in 2013 to 637,650 in 2017 [20]. Likewise, in China, the volume grew from 284,936 in 2010 to 915,256 in 2018, with an annual growth rate ranging from 10 to 20% [21]. Many patients who undergo revascularization are at risk of experiencing recurrent revascularization due to lesion progression or in-stent stenosis, particularly those with type 2 diabetes mellitus (T2DM). Hence, it is crucial to identify early on the patients with a high residual risk for poor prognosis, as this would facilitate accurate risk stratification and allow for personalized secondary prevention strategies to reduce the likelihood of future recurrent revascularization.

Insulin resistance, a pivotal hormone that regulates cellular metabolism, is closely linked to the development and progression of atherosclerotic plaque [8]. Previous research has shown that insulin resistance, as measured by the homeostasis model assessment of insulin resistance (HOMA-IR), may be a causative factor for elevated levels of prothrombotic factors, proinflammatory markers, and reactive oxygen species (ROS) [7, 22]. These in turn trigger oxidative stress, inflammation, and subsequent endothelial dysfunction associated with atherosclerosis [5, 6, 8]. Furthermore, dyslipidemia resulting from insulin resistance, characterized by increased plasma triglyceride levels, decreased levels of high-density lipoprotein (HDL), and the presence of small dense low-density lipoproteins (sdLDL) [23–25], also contributes to the formation of atherosclerotic plaques [8]. Consequently, the TyG index, considered as an affordable, practical, and reproducible surrogate marker of insulin resistance in comparison to the hyperinsulinemic-euglycemic clamp test and HOMA-IR [10], has shown associations with the complexity of coronary artery lesions in several observational clinical studies [26, 27].

Besides, multiple clinical evidence has also demonstrated that the TyG index was not only positively correlated with the rapid progression of coronary atherosclerosis [28, 29] and calcification [30] but was also an independent risk factor for poor long-term prognosis in patients with coronary heart disease, including acute myocardial infarction (AMI) [31], chronic total occlusion (CTO) [32], premature coronary artery disease [33], and patients with or without diabetes mellitus [11, 12]. Furthermore, Zhu et al. indicated that an elevated TyG index was independently and positively associated with ISR in patients with ACS after PCI with drug-eluting stents [34]. However, few investigators have focused on the relationship between the TyG index and the risk of recurrent revascularization in diabetic patients after PCI. In line with previous studies, we demonstrated that a higher TyG index was independently associated with recurrent revascularization resulting from the progression of lesion or ISR. Further analysis revealed that the progression of lesions was the main factor contributing to recurrent revascularization in the present cohort, rather than in-stent restenosis.

Furthermore, recent research has demonstrated that the triglyceride glucose (TyG) index can be utilized to optimize early risk stratification in patients with coronary artery disease following PCI. The combination of the TyG index and the GRACE score provide a significant improvement in prediction of adverse cardiovascular outcomes in patients with non-ST-segment elevation acute coronary syndrome (NSTEMI) [35] or acute coronary syndrome (ACS) [36] undergoing PCI in terms of the C-statistic value, NRI and IDI. The present study additionally revealed that incorporating the TyG index into the established risk model improved model discrimination and risk reclassification capabilities, as indicated by a C-index of 0.759 (95% CI 0.724–0.792, P < 0.01), cNRI > 0 of 0.170 (95% CI 0.023–0.287, P < 0.05), and IDI of 0.024 (95% CI 0.009–0.039, P = 0.002). Conversely, the GRACE score fails to consider factors such as inflammation, cardiometabolic risk factors, nutritional status, and other indicators that could significantly impact the long-term prognosis of patients with acute coronary syndrome (ACS), and the existing risk prediction models for these events also possess inherent limitations [16, 37]. Our findings suggest that incorporating cardiometabolic risk factors, like the TyG index, into risk assessment models may aid in the identification of high-risk diabetes mellitus patients for recurrent revascularization after PCI.

Moreover, despite intensive lipid-lowering regimens comprising statins in combination with ezetimibe and/or proprotein convertase subtilisin/kexin type 9 (PCSK9) inhibitors, there remains a risk of cardiovascular adverse events in certain populations, indicating the presence of residual cardiovascular risk [38]. Elevated levels of triglycerides (TG) and insulin resistance play significant roles in the persistence of cardiovascular risk following intensive lipid-lowering therapy [39]. Controlling TG levels is a crucial aspect of managing residual risk. Notably, the REDUCE-IT study demonstrated that oral icosapent ethyl (IPE) was associated with a 25% reduction in the risk of the primary endpoint (HR 0.75; 95% CI 0.68–0.83; P < 0.001), as well as a 26% reduction in the risk of secondary endpoint events (95% CI 0.65–0.83; P < 0.001) [40]. Furthermore, several recent trials have shown that interventions targeting the improvement of insulin resistance, such as glucagon-like peptide-1 analogues (GLP-1) [41], sodium–glucose cotransporter 2 inhibitors (SGLT-2i) [42], and thiazolidinediones (TZDs) [43], have the potential to further mitigate the risk of adverse cardiovascular events in both diabetic and non-diabetic individuals.

## Limitations

However, there are some limitations that merit consideration in this study. First, this is a single-center, observational study with a relatively small sample size. Consequently, the results of this research should be interpreted with caution and confirmed through additional studies conducted in different settings. Additionally, the TyG index was evaluated using blood samples collected after admission. It is important to investigate whether fluctuations in the TyG index, which may be influenced by intensive lipid-lowering therapy or insulin sensitizer, are associated with recurrent revascularization in patients with T2DM undergoing PCI. Furthermore, this study did not enroll patients who did not receive revascularization due to some social factors, as well as including some asymptomatic patients who potentially needed revascularization, which may lead to potential bias. Moreover, this study only enrolled patients with T2DM undergoing PCI, and as a result, the applicability of the findings to non-diabetic patients is limited. Finally, the study lacks true external validation. The transportability and generalizability of the results should be validated in future patients.

## Conclusion

The study provided evidence of an independent association between the TyG index and the risk of recurrent revascularization in patients with T2DM after PCI. Notably, lesion progression emerged as the primary contributor to recurrent revascularization instead of in-stent restenosis. Moreover, after accounting for competitive risk events, the TyG index continues to be a significant risk factor for recurrent revascularization. The inclusion of the TyG index in an established model improves the predictive value for recurrent revascularization. These findings have implications for precisely stratifying risk and optimizing secondary prevention and follow-up measures for diabetic patients. Importantly, implementing modern therapies that target insulin resistance may potentially delay the advancement of coronary lesions and further decrease the likelihood of recurrent revascularization after PCI.

### Supplementary Information

**Additional file 1: Table S1.** Univariate competing risk analysis and Cox regression analysis for predicting recurrent revascularization after PCI. **Table S2.** Comparisons of the area under the ROC curves of the TyG index, FBG, HbA1c and TG. **Table S3.** ROC curve analysis of the TyG index, HbA1c, TG and FBG for recurrent revascularization. **Table S4.** The model performance estimated by internal bootstrap validation method. **Figure S1.** Flow chart. **Figure S2.** Reasons for unplanned revascularization.

## Data Availability

The datasets used and/or analyzed in the study are available from the corresponding author upon reasonable request.
