# Supplementary material for: Triglyceride-glucose index is associated with recurrent revascularization in patients with type 2 diabetes mellitus after percutaneous coronary intervention
Source: Cardiovasc Diabetol. 2023 Oct 21;22:284. doi: 10.1186/s12933-023-02011-2 (PMC10590524; doi:10.1186/s12933-023-02011-2)
Supplement: Supplementary file 1 — Additional file 1: Table S1. Univariate competing risk analysis and Cox regression analysis for predicting recurrent revascularization after PCI. Table S2. Comparisons of the area under the ROC curves of the TyG index, FBG, HbA1c and TG. Table S3. ROC curve analysis of the TyG index, HbA1c, TG and FBG for recurrent revascularization. Table S4. The model performance estimated by internal bootstrap validation method. Figure S1. Flow chart. Figure S2. Reasons for unplanned revascularization. [file 12933_2023_2011_MOESM1_ESM.doc]

**Table S1. Univariate competing risk analysis and Cox regression analysis for predicting recurrent revascularization after PCI**

| Variables | Univariate competing risk analysis | | | Univariate Cox regression analysis | | |
| --- | --- | --- | --- | --- | --- | --- |
|  | SHR | 95%CI | P | HR | 95%CI | P |
| Age, years | 1.0218 | 0.9987 to 1.0455 | 0.065 | 1.0237 | 1.0002 to 1.0477 | 0.048 |
| Female | 0.9132 | 0.5401 to 1.5439 | 0.735 | 0.9253 | 0.5453 to 1.5704 | 0.774 |
| BMI, kg/m2 | 0.9194 | 0.8537 to 0.9903 | 0.027 | 0.9170 | 0.8450 to 0.9952 | 0.038 |
| Smoking | 1.2173 | 0.7472 to 1.9833 | 0.430 | 1.2031 | 0.7366 to 1.9651 | 0.460 |
| Previous PCI | 1.5077 | 0.7844 to 2.8980 | 0.218 | 1.4976 | 0.7623 to 2.9421 | 0.241 |
| Hypertension | 1.0813 | 0.6077 to 1.9241 | 0.790 | 1.0981 | 0.6155 to 1.9592 | 0.751 |
| SBP, mmHg | 0.9989 | 0.9883 to 1.0095 | 0.834 | 0.9984 | 0.9874 to 1.0095 | 0.778 |
| Heart rate, bpm | 1.0104 | 0.9960 to 1.0250 | 0.158 | 1.0116 | 0.9954 to 1.0281 | 0.161 |
| BNP, pg/ml | 1.0000 | 0.9997 to 1.0003 | 0.885 | 1.0001 | 0.9997 to 1.0005 | 0.708 |
| cTnT, pg/ml | 0.9999 | 0.9998 to 1.0001 | 0.368 | 0.9999 | 0.9998 to 1.0001 | 0.454 |
| Serum creatinine, µmol/L | 1.0017 | 1.0009 to 1.0025 | ＜0.001 | 1.0017 | 1.0007 to 1.0027 | 0.001 |
| Uric acid, µmol/L | 1.0005 | 0.9983 to 1.0027 | 0.656 | 1.0008 | 0.9987 to 1.0029 | 0.455 |
| HbA1c, % | 1.0002 | 0.9975 to 1.0028 | 0.905 | 0.9757 | 0.8478 to 1.1229 | 0.731 |
| FBG, mmol/L | 1.0497 | 0.9860 to 1.1176 | 0.129 | 1.0555 | 0.9913 to 1.1238 | 0.092 |
| TC, mmol/L | 1.0923 | 0.8815 to 1.3536 | 0.420 | 1.0945 | 0.9063 to 1.3218 | 0.349 |
| TG, mmol/L | 1.1815 | 0.9705 to 1.4384 | 0.097 | 1.1780 | 0.9267 to 1.4974 | 0.181 |
| HDL-C, mmol/L | 1.5890 | 0.6846 to 3.6882 | 0.281 | 1.5964 | 0.7156 to 3.5614 | 0.253 |
| LDL-C, mmol/L | 1.0758 | 0.7792 to 1.4854 | 0.657 | 1.0809 | 0.8196 to 1.4256 | 0.582 |
| AMI | 1.4207 | 0.8734 to 2.3110 | 0.157 | 1.4131 | 0.8650 to 2.3087 | 0.167 |
| bSS | 1.0612 | 1.0425 to 1.0802 | <0.001 | 1.0643 | 1.0404 to 1.0887 | <0.001 |
| rSS | 1.0807 | 1.0541 to 1.1079 | <0.001 | 1.0821 | 1.0520 to 1.1130 | <0.001 |
| Number of stents | 1.1435 | 0.9157 to 1.4280 | 0.237 | 1.1331 | 0.8975 to 1.4307 | 0.293 |
| Length of stents, mm | 1.0042 | 0.9968 to 1.0118 | 0.267 | 1.0039 | 0.9961 to 1.0117 | 0.327 |
| Tyg index | 1.6861 | 1.1723 to 2.4250 | 0.005 | 1.7196 | 1.1548 to 2.5606 | 0.008 |
| LVEF, % | 0.9889 | 0.9655 to 1.0129 | 0.363 | 0.9850 | 0.9618 to 1.0089 | 0.216 |
| Statins | 0.8057 | 0.1862 to 3.4860 | 0.773 | 0.7397 | 0.1803 to 3.0351 | 0.676 |
| β-blockers | 0.6292 | 0.3810 to 1.0391 | 0.070 | 0.6230 | 0.3769 to 1.0296 | 0.065 |
| Diuretics | 1.2871 | 0.7337 to 2.2576 | 0.379 | 1.4119 | 0.8013 to 2.4875 | 0.233 |
| ACEI/ARB | 1.0262 | 0.6281 to 1.6767 | 0.918 | 1.0331 | 0.6327 to 1.6869 | 0.897 |
| Insulin | 2.1164 | 1.2800 to 3.4992 | 0.003 | 2.1396 | 1.2935 to 3.5391 | 0.003 |

BMI, body mass index; PCI, percutaneous coronary intervention; SBP, systolic blood pressure; BNP, brain natriuretic peptide; FBG, fasting blood glucose; TG, triglyceride; TC, total cholesterol; HDL-C, high density lipoprotein cholesterol; LDL-C, low density lipoprotein cholesterol, LVEF, left ventricular ejection fraction; AMI, acute myocardial infarction, ACEI/ARB, angiotensin converting enzyme inhibitor/angiotensin receptor blocker; TyG index, the triglyceride–glucose index; bSS, baseline SYNTAX score; rSS, residual SYNTAX score; SHR, subdistribution hazard ratio; HR, hazard ratio; CI, confidence interval.

Table S2. Comparisons of the area under the ROC curves of the TyG index, FBG, HbA1c and TG

| variable | δAUC | 95% CI | z | p value |
| --- | --- | --- | --- | --- |
| TyG index vs. FBG | 0.0971 | 0.0299 to 0.164 | 2.830 | 0.0046 |
| TyG index vs. TG | 0.0243 | -0.0286 to 0.0772 | 0.901 | 0.3675 |
| TyG index vs. HbA1c | 0.116 | 0.00326 to 0.229 | 2.016 | 0.0438 |

ROC curve, ﻿receiver operating characteristic curve; AUC, aera under the curve; TyG index, triglyceride–glucose index; FBG, fasting blood glucose; TG, triglyceride.

**Table S3. ROC curve analysis of the TyG index, HbA1c, TG and FBG for recurrent revascularization**

| variable | AUC | 95% CI | specificity | sensitivity | z statistic | P value |
| --- | --- | --- | --- | --- | --- | --- |
| TyG index | 0.636 | 0.566 to 0.707 | 50.09 | 78.12 | 3.793 | < 0.001 |
| HbA1c | 0.520 | 0.448 to 0.592 | 51.67 | 57.81 | 0.547 | 0.585 |
| TG | 0.612 | 0.544 to 0.680 | 34.27 | 82.81 | 3.217 | 0.001 |
| FBG | 0.539 | 0.466 to 0.612 | 22.85 | 89.06 | 1.053 | 0.292 |

ROC curve, ﻿receiver operating characteristic curve; AUC, aera under the curve; TyG index, triglyceride–glucose index; FBG, fasting blood glucose; TG, triglyceride.

**Table S4**. The model performance estimated by internal bootstrap validation method

|  | C-index(95%CI) | P value | Bias-corrected C-index(95%CI) | P value |
| --- | --- | --- | --- | --- |
| Established risk model | 0.739(0.703, 0.773) | ＜0.01 | 0.747(0.692, 0.793) | ＜0.01 |
| Established risk Model+ TG | 0.750(0.715, 0.783) | ＜0.01 | 0.757(0.705, 0.802) | ＜0.01 |
| Established risk Model+ HbA1c | 0.745(0.709, 0.778) | ＜0.01 | 0.753(0.697, 0.800) | ＜0.01 |
| Established risk Model+ FBG | 0.740(0.704, 0.774) | ＜0.01 | 0.751(0.703, 0.797) | ＜0.01 |
| Established risk Model +TyG | 0.759(0.724, 0.792) | ＜0.01 | 0.769(0.718, 0.812) | ＜0.01 |

TyG index, the triglyceride-glucose index; TG, triglyceride; FBG: fasting blood glucose; Established risk model included age, BMI, Scr, bSS, rSS and insulin.

**
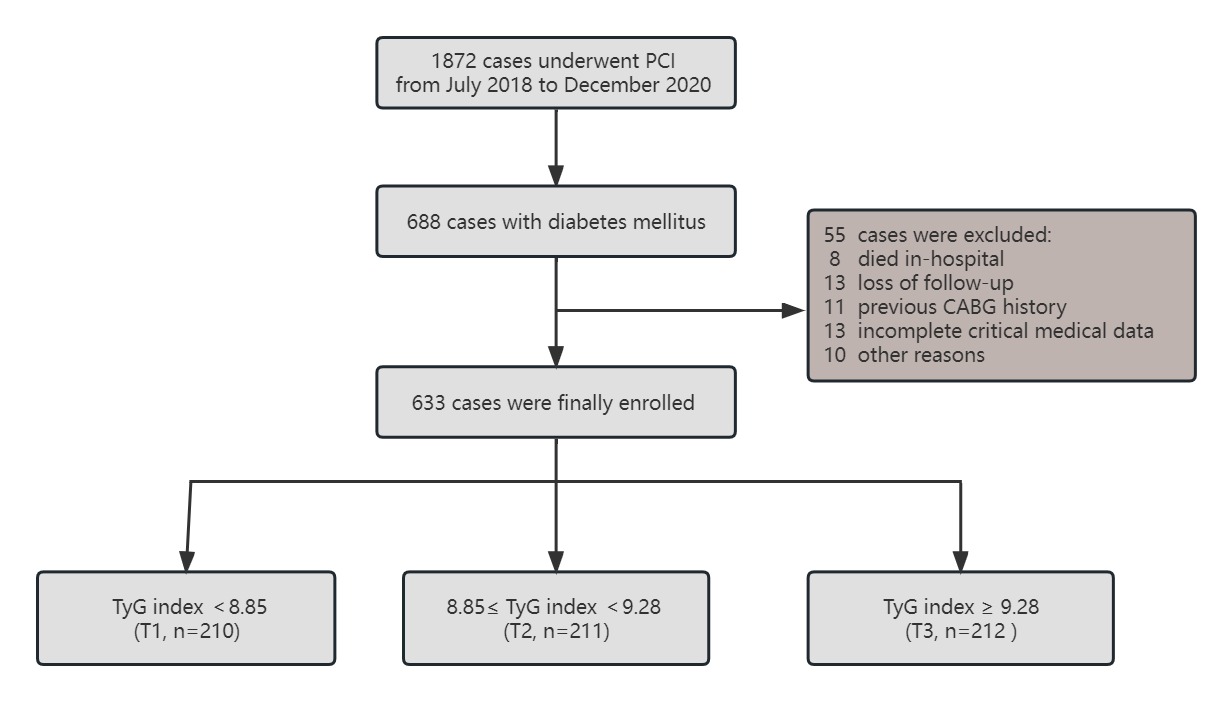
**

**FigureS1. Flow chart**

From July 2018 to December 2020, a total of 1,872 patients received percutaneous coronary intervention (PCI), of which 688 patients had diabetes in this study. 55 patients were excluded from the study. These exclusions comprised 8 patients who died in the hospital, 13 patients who were lost to follow-up, 11 patients with previous coronary artery bypass grafting (CABG), 13 patients with missing critical data, and 10 patients for other reasons. Thus, a final sample size of 633 patients was included. The patients enrolled were divided into three groups in accordance with tertiles of the TyG index [T1 (TyG index＜8.85), T2 (8.85≤TyG index＜9.28), and T3 (TyG index≥9.28)]. CABG: coronary artery bypass grafting.


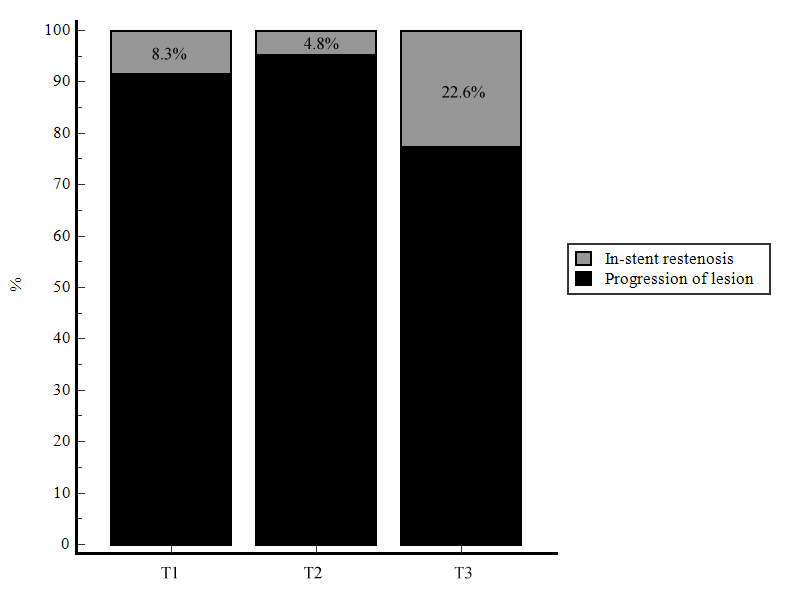


Figure S2. Reasons for unplanned revascularization

Amongst the recurrent revascularization events (64 cases) comprised of 55 cases of lesion progression and 9 cases of in-stent restenosis. The proportion of lesion progression surpasses that of in-stent restenosis among all three groups. T1: TyG index＜8.85; T2 : 8.85≤TyG index＜9.28; T3: TyG index≥9.28).
